# Colorectal cancer and adenoma screening using urinary volatile organic compound (VOC) detection: early results from a single-centre bowel screening population (UK BCSP)

**DOI:** 10.1007/s10151-019-01963-6

**Published:** 2019-04-15

**Authors:** E. Mozdiak, A. N. Wicaksono, J. A. Covington, R. P. Arasaradnam

**Affiliations:** 1 University Hospitals Coventry and Warwickshire, Coventry, UK; 2 School of Engineering, The University of Warwick, Coventry, UK

**Keywords:** Colorectal cancer, Bowel cancer screening, Volatile organic compounds, Urinary biomarkers

## Abstract

**Background:**

The United Kingdom (UK) bowel cancer screening programme has reduced mortality from colorectal cancer (CRC), but poor uptake with stool-based tests and lack of specificity of faecal occult blood testing (FOBT), has prompted investigation for a more suitable screening test. The aim of this study was to investigate the feasibility of a urinary volatile organic compounds (VOC)-based screening tool for CRC.

**Methods:**

The urine from FOBT-positive patients was analysed using field asymmetric ion mobility spectrometry (FAIMS) and gas chromatography coupled with ion mobility spectrometry (GC–IMS). Data were analysed using a machine learning algorithm to calculate the test accuracy for correct classification of CRC against adenomas and other gastrointestinal pathology.

**Results:**

One hundred and sixty-three patients were enrolled in the study. Test accuracy was high for differentiating CRC from control: area under the curve (AUC) 0.98 (95% CI 0.93–1) and 0.82 (95% CI 0.67–0.97) using FAIMS and GC–IMS respectively. Correct classification of CRC from adenoma was high with AUC range 0.83–0.92 (95% CI 0.43–1.0). Classification of adenoma from control was poor with AUC range 0.54–0.61 (95% CI 0.47–0.75) using both analytical modalities.

**Conclusions:**

CRC was correctly distinguished from adenomas or no bowel pathology using urinary VOC markers, within the bowel screening population. This pilot study demonstrates the potential of this method for CRC detection, with higher test uptake and superior sensitivity than FOBT. In addition, this is the first application of GC–IMS in CRC detection which has shown high test accuracy and usability.

**Electronic supplementary material:**

The online version of this article (10.1007/s10151-019-01963-6) contains supplementary material, which is available to authorized users.

## Introduction

Colorectal cancer (CRC) is a leading cause of cancer-related deaths in the western world [1, 2]. It represents the fourth most common cancer in England and Wales and is the second leading cause of cancer-related deaths [1].

The United Kingdom (UK) bowel cancer screening programme (UK BCSP) was implemented in 2007 following a series of randomised controlled trials that demonstrated a reduction in mortality from CRC due to screening [3–6]. However, the current guaiac FOBT has a number of well-documented disadvantages including low uptake (around 60%), low specificity and the test interpretation has the potential for human error [7, 8]. The introduction of the faecal immunochemical test (FIT) as the first-line bowel screening test in the UK is anticipated within the next year and is associated with improved uptake by as much as 25% and higher sensitivity [7, 9]. However, overall test accuracy could be improved further, to detect more CRC cases via screening and prevent unnecessary colonoscopies.

There is a dearth of research on new biomarkers for CRC detection. One area that has gained momentum over the past decade is the use of volatile organic compounds (VOCs). VOCs are organic chemicals that have a high vapour pressure at room temperature, i.e. that evaporate or sublimate readily under ambient conditions. They can be captured from a variety of body mediums and have been shown to alter in different disease states [10, 11].

Applying the use of gas phase markers to diagnose CRC is a rapidly expanding area, but it involves highly complex sample and data analysis. Detection of VOCs in CRC using a variety of different mediums has been investigated in small-scale studies [12–14]. Few have used urine analysis or focussed on the asymptomatic BCSP cohort. There are currently no VOC-based tests established in the clinical setting for any disease [15–18].

Using urine for VOC detection has the advantage that urine is simple to collect and is readily available and collection is associated with high patient acceptability. It is also easy to store and shows stability in the medium term [19]. These factors make it an ideal focus for the development of a screening tool as an alternative to a faeces-based test.

This study aims to establish whether urinary VOC markers can be utilised as a tool for detecting CRC and adenomas within the UK BCSP. Sample analysis was conducted using field asymmetrical ion mobility spectrometry (FAIMS), which has been employed in a small number of studies before [20–23]. In addition, a new analytical modality for disease detection called gas chromatography coupled with ion mobility spectrometry (GC–IMS) was employed.

## Materials and methods

All enrolled patients were recruited from the Coventry and Warwickshire University Hospitals between April 2015 and November 2016. Regional ethical approval was granted by the Warwickshire Research and Development Department and Warwickshire Ethics Committee 09/H1211/38. Informed consent was obtained from the individual participants that took part in the study. This study was approved by the bowel cancer screening research committee only to approach those that had a positive FOBT test.

### Patient recruitment

Patients were recruited from the nurse-led BCSP clinics following a positive FOBT result. Consent and urine sample collection were carried out at the clinic prior to bowel preparation administration. A total of 181 patients were invited to participate and 163 consented to provide samples for the final analysis.

### Sample collection and storage

Two 20 ml samples of urine were collected. Samples were immediately transferred to − 20 °C storage and then to − 80 °C within 24 h for long-term storage. Diagnostic outcome data were collected from the colonoscopy or computed tomography (CT) colonography result, histology was confirmed from the pathology report.

### Sample analysis

Samples were analysed using the Lonestar FAIMS instrument (Owlstone, UK) and the Silox GC–IMS (Imspex, UK). FAIMS is a gas detection technology that separates chemical ions, within a complex mixture of VOCs, based on their mobility/movement in high electrical fields. This technique has been described in detail before. Sampling methods can be found in previous publications by our group [12, 23]. A detailed schematic for FAIMS analysis is found in Online Resource 1.

**Fig. 1:**
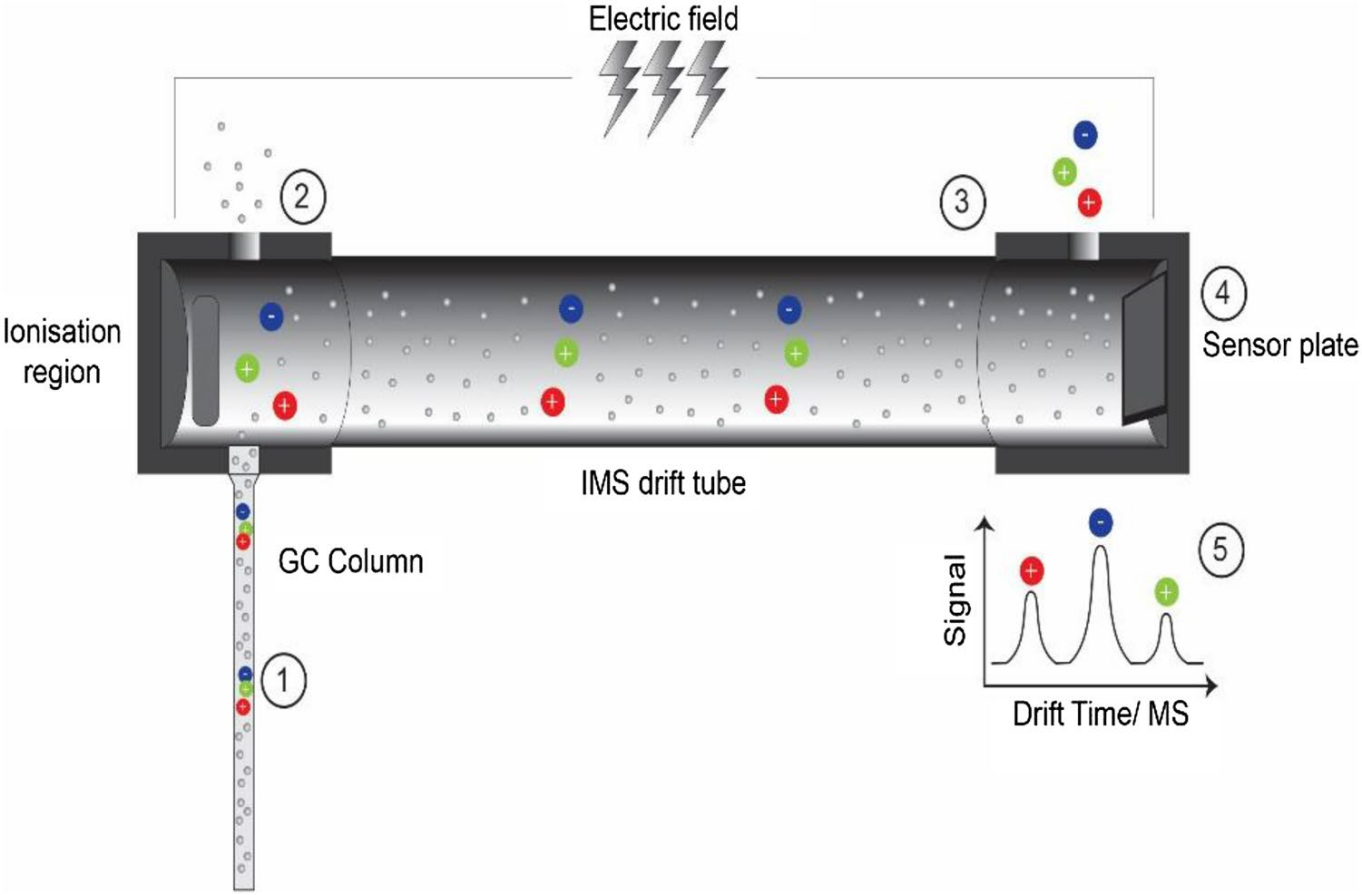
Schematic of the separation process and ion detection using gas chromatography–ion mobility spectrometry (GC–IMS). (1) Sample passes through the gas column where initial separation occurs. (2) The discrete compounds are consecutively fed into the ionisation chamber where ionisation occurs. (3) Ions pass through the drift tube at varying speeds dependent on their mobility. (4) Ions hit the sensor plate and are detected. (5) Ion peaks are calculated based on drift time

**Fig. 2:**
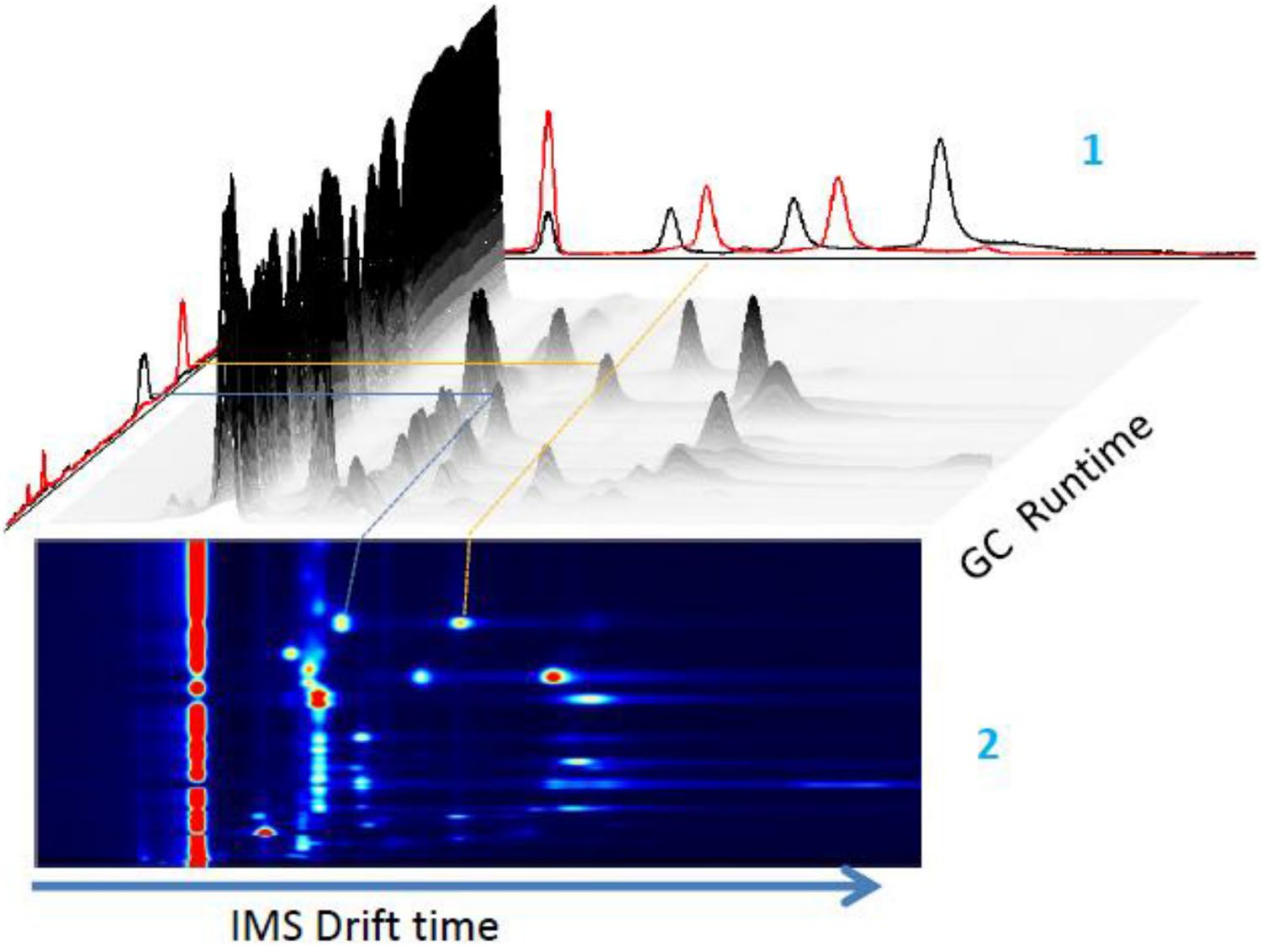
Three-dimensional representation of gas chromatography (GC) data output with corresponding ion mobility spectrometry (IMS) chromatogram. (1). Single IMS spectra data is combined with GC run time peaks. (2) Heatmap corresponding to GC–IMS peaks (yellow and blue lines) (Image adapted with permission from Impsex, UK). Data output is twofold: gas chromatography (GC) gives peaks representing retention time as the ions pass thorough the column. This is coupled with ion mobility spectrometry (IMS) data, based on the mobility of the ions as they pass through the drift tube and hit the sensor. The culmination of this two-phase analysis is represented as an IMS chromatogram which incorporates millions of data points in a heatmap (Fig. 2). These data points are subject to very similar statistical analysis as is applied to the Lonestar data

GC–IMS involves a two-stage analytical process. The first stage uses a gas chromatograph to separate VOCs based on their interaction with a coated capillary column. Then these VOCs are detected as they elude from the column using a drift-tube ion mobility spectrometer, where the time taken for chemical ions to travel along a tube (against a flow of buffer gas) is measured. Chemical ions of different sizes take different lengths of time to travel along the tube and this can be used to separate chemical species. As this has not previously been described sample analysis is outlined here:

A 5 ml urine sample is aliquoted into a 20 ml glass vial and sealed with a crimp lid. A 21 g needle is attached to the GC–IMS input port. The needle with attached port is inserted into the sample headspace 1 cm above the urine. The needle is held in place for 20 s to allow for vapour aspiration. The total run time is 5 min per sample. The carrier gas flow rate is 150 ml/min and sample flow rate through the instrument is 20 ml/min. The sample heating was carried out in accordance with manufacturer instructions to a maximal level of 80 °C. A schematic for the GC–IMS detection process is shown in Fig. 1 and the three-dimensional data output with corresponding heat map is shown in Fig. 2.

### Statistical analysis

The analysis of data created through FAIMS and GC–IMS analysis employs machine learning methods. In brief, they involve the construction of computerised algorithms that can learn from and make predictions on the output data from the instrument. The algorithms build a prediction model from a training set of known cases and use this knowledge to predict output decisions (diagnoses) on unknown cases. Five classification models were used; each dataset was compared with each model to find the most accurate for each specific set of samples. This form of analysis produces receiver operating characteristic (ROC) curves with area under the curve (AUC), sensitivity and specificity values calculated from the coordinates of the ROC plots. A schematic of the analysis pipeline is provided in Online Resource 2. All statistical analyses were carried out using the verification package in R studio (R Foundation for Statistical Computing, Vienna, Austria).

As CRC numbers were small within the screening population (incidence of 8–10%), a balancing technique was applied to the data to fairly match the non-CRC samples with the same number of CRC samples and avoid bias from an unbalanced control group. Balancing involved the well-described synthetic minority over-sampling technique (SMOTE), where artificially generated points are plotted to represent the control group as a whole and is used to provide a more fair representation [24].

## Results

### FAIMS analysis

A total of 163 samples were analysed. 93 (57%) were from males, median age of patients was 67 years, 12 (7.4%) were current smokers. 41 (25.4%) were ex-smokers and 109 (67.2%) had never smoked. Patients were grouped into categories according to diagnosis for analysis. Diagnostic outcomes for study participants are listed in Table 1.

**Table 1:** Diagnostic outcomes for study participants and distribution of CRC by site (total of 13 cancer sites as one patient had a synchronous tumours)

Diagnosis	Number (%)	
Cancer	Total	12 (7.6)
	Rectum	4 (2.4)
	Sigmoid	4 (2.4)
	Descending colon	0
	Transverse colon	1 (0.58)
	Ascending colon	2 (1.17)
	Cecum	2 (1.17)
Adenoma	Total	80 (49.1)
	High	17(10.5)
	Intermediate	36 (21.1)
	Low	27 (17.5)
Diverticular disease		14 (8.2)
Normal		37 (19.3)
Haemorrhoids		5 (2.9)
Other		14 (8.2)^
Excluded		8 (4.7)*

*1 not fit enough for investigations, 7 declined investigations

^Inflammatory bowel disease: *n* = 7, rectal telangiectasia: *n* = 2, rectal ulcer: *n* = 1, radiation proctitis: *n* = 1, inflammatory pseudopolyp: *n* = 1, non-specific sigmoid inflammation: *n* = 1, ischaemic sigmoid stricture: *n* = 1

**Fig. 3:**
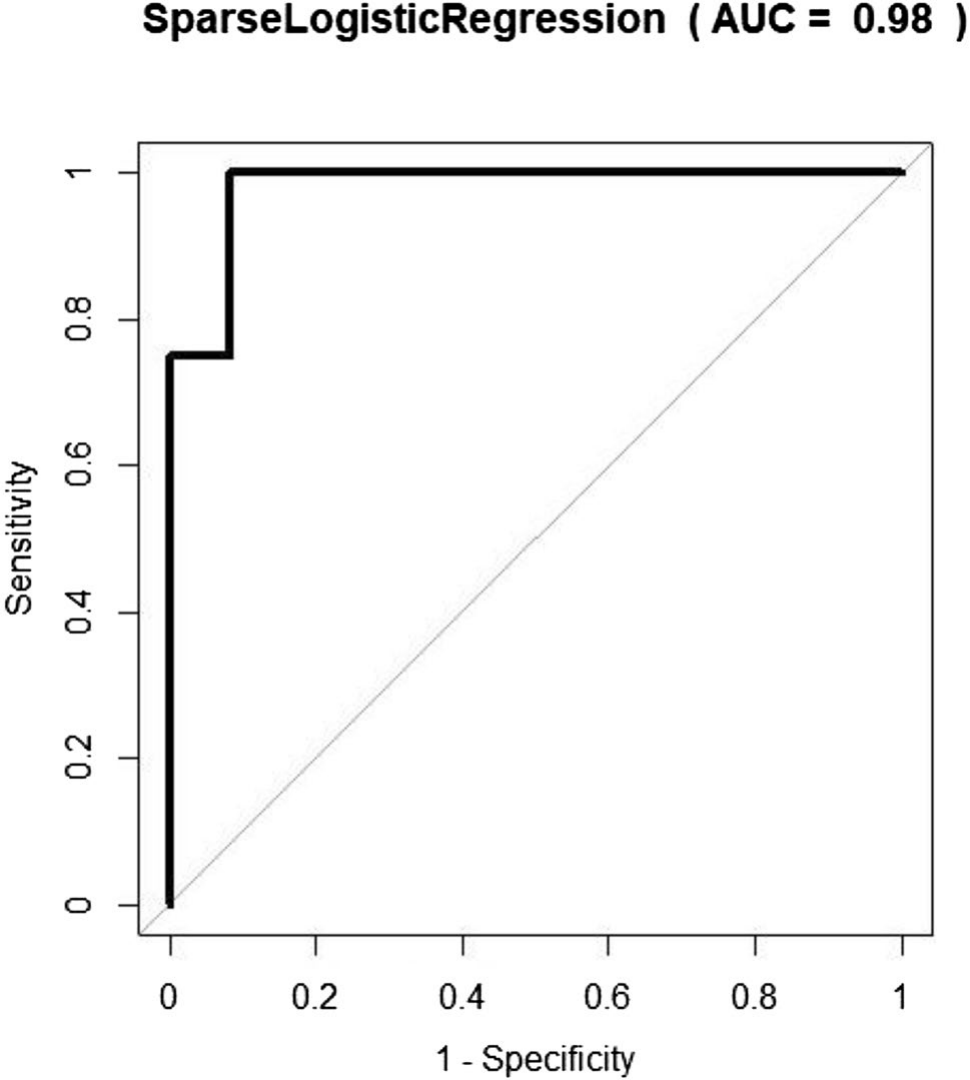
Receiver operating characteristic (ROC) curve for classification of colorectal cancer (CRC) vs normal in bowel cancer screening programme (BCSP) patients (balanced) using the sparse logistic regression classifier using field asymmetric waveform ion mobility spectrometry(FAIMS)

Group (a) CRC vs normal control demonstrated the highest degree of separation with AUC 0.98 (95% CI 0.93–1.0) with 12 patients in each group. The corresponding ROC curve is shown in Fig. 3. Sensitivity and specificity were also high: 1.0 (95% CI 0.74–1) and 0.92 (95% 0.62–1), respectively (Table 2).

**Table 2:** Classification of BCSP study participants by outcome using FAIMS

Group	AUC	Sensitivity	Specificity	PPV	NPV
a) CRC (12) vs normal (12)	0.98 (0.93–1)	1 (0.74–1)	0.92 (0.62–1)	0.92	1
b) CRC + all adenomas (93) vs normal (37)	0.64 (0.54–0.74)	0.48 (0.38–0.59)	0.89 (0.75–0.97)	0.92	0.41
c) CRC + high-risk adenomas (30) vs normal (37)	0.62 (0.48–0.76)	0.57 (0.37–0.75)	0.68 (0.5–0.82)	0.59	0.66
d) CRC + high-risk adenomas (30) vs other (70)	0.6 (0.47–0.73)	0.47 (0.28–0.66)	0.80 (0.68–0.89)	0.52	0.76
e) CRC + all adenomas (93)vs other (70)	0.56 (0.47–0.65)	0.91 (0.84–0.96)	0.25 (0.15–0.38)	0.64	0.67
f) Non-CRC (113) vs normal (37)	0.61 (0.51–0.71)	0.56 (0.46–0.65)	0.68 (0.5–0.82)	0.83	0.35
g) CRC (12) vs Adenoma (7) (hr)	0.92 (0.77–1)	0.83 (0.52–0.98)	1 (0.59–1)	1	0.78
h) CRC (12) vs Adenoma (12) (ir)	0.84 (0.67–1)	0.83 (0.52–0.98)	0.75 (0.43–0.95)	0.77	0.82
i) CRC (12) vs Adenoma (12) (lr)	0.83 (0.66–1)	0.75 (0.43–0.95)	0.92 (0.62–1)	0.90	0.79

Using sparse logistic regression and Gaussian process

Corresponding 95% CIs are stated in brackets. Numbers in brackets in group column denote sample number

*BSCP* bowel cancer screening programme, *FAIMS* field asymmetric waveform ion mobility spectrometry, *CRC* Colorectal cancer

In groups (b–e) CRC was grouped with adenomas and showed only modest AUC, sensitivity and specificity results, when attempting to classify groups according to diagnosis. The most accurate classification of the adenoma groups was seen in (b) CRC + all adenomas vs normal control; here sensitivity was low at 0.48 (95% CI 0.38–0.59) but specificity was high at 0.89 (95% CI 0.75–0.97). In (f) when CRC was excluded, separation was low with sensitivity 0.56 (95% CI 0.46–0.65).

A further set of analyses were carried out to investigate the classification of the adenoma groups in more depth and to compare CRC with three categories of adenoma: (g) high risk, (h) intermediate risk and (i) low risk, according to the BSG guidelines [25], the results are displayed in Table 1. High sensitivity was demonstrated when each adenoma group was compared with CRC. The most accurate overall classification was seen in CRC vs high-risk adenoma with a sensitivity of 0.83 (95% CI 0.52–0.98) and specificity of 1 (95% CI 0.59–1).

### GC–IMS analysis

One hundred and nine patient samples were analysed using the Silox GC–IMS instrument. Five comparator groups were devised according to outcome (Table 3).

**Fig. 4:**
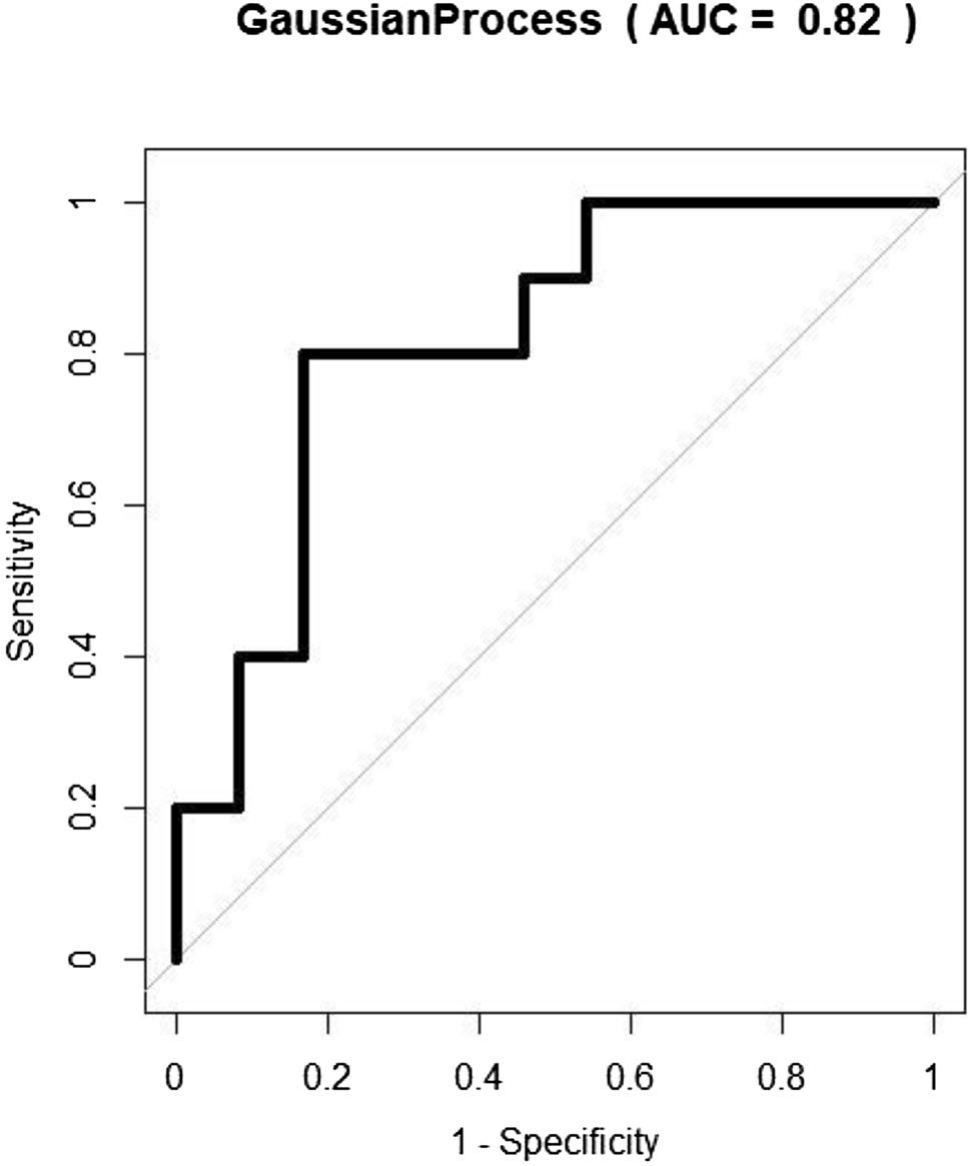
Receiver operating characteristic (ROC) curve for classification of colorectal cancer (CRC) vs normal using GC–IMS. [Gaussian process (GP) classifier]

As with the analysis using FAIMS, when comparing CRC vs normal control (group a) there was a high degree of separation with a sensitivity of 0.80 (95% CI 0.44–0.97) and specificity of 0.83 (95% CI 0.63–0.95). The corresponding ROC curve is seen in Fig. 4. CRC vs other diagnoses also had a high sensitivity of 1.0 (95% CI 0.66–1), however, specificity dropped to 0.57 (95% CI 0.34–0.78). When CRC samples were grouped with adenomas and compared with other groups (those with any diagnosis other than CRC or adenoma) the sensitivity dropped to a modest level of 0.71 (95% CI 0.58–0.81) with sensitivity 0.55 (0.39–0.70). Adenomas vs normal control showed a low level of separation, with a sensitivity of only 0.58 (95% CI 0.44–0.71) and specificity 0.62 (95% CI 0.41–0.81) (Table 3).

**Table 3:** Classification of BCSP study participants using GC–IMS using Gaussian process or support vector machine

Group	AUC	Sensitivity	Specificity	PPV	NPV
CRC (10) vs normal (24)	0.82 (0.67–0.97)	0.80 (0.44–0.97)	0.83 (0.63–0.95)	0.67	0.91
CRC + high-risk adenomas (23) vs normal(24)	0.53 (0.36–0.70)	0.48 (0.27–0.69)	0.67 (0.45–0.84)	0.58	0.57
CRC (10) vs other (20)	0.77 (0.60–0.94)	1 (0.66-1)	0.57 (0.34–0.78)	0.5	1
CRC + all adenomas (65) vs other (42)	0.61 (0.49–0.72)	0.71 (0.58–0.81)	0.55 (0.39–0.70)	0.71	0.55
All adenomas (55) vs normal (24)	0.61 (0.47–0.75)	0.58 (0.44–0.71)	0.62 (0.41–0.81)	0.78	0.39

Corresponding 95% CI are in brackets. Study numbers are stated in the group column in brackets

*BSCP* bowel cancer screening programme, *GC–IMS* gas chromatography coupled with ion mobility spectrometry, *AUC* area under the curve, *CRC* colorectal cancer, *PPV* positive predictive value, *NPV* negative predictive value

## Discussion

This study explored the feasibility of a urinary VOC-based test in the detection of CRC within the screening population that tested positive for faecal occult blood. The results, applying two different technologies—FAIMS and GC–IMS show consistency. Ability to distinguish CRC from normal control was high with AUC 0.98, sensitivity of 1.0 and specificity of 0.92. Ability to distinguish CRC from low-, intermediate- and high-risk adenomas was high with test accuracy ranging from 0.83 to 0.92 using FAIMS. Similarly, ability to distinguish CRC from normal control was high with AUC 0.82, sensitivity of 0.80 and specificity of 0.83 using GC–IMS.

CRC-specific VOCs are thought to occur via genetic and protein changes that cause peroxidation of the cell membrane [26]. In addition, there is an increase in reactive oxygen species within the cancer cell and alterations in the microbiome have a direct effect on the VOCs [27–30]. How distinct these changes are in the CRC group compared with other disease groups is yet to be fully elucidated.

The separation of CRC from normal controls was high, yet when CRC cases were grouped with adenomas the accuracy dropped significantly. This suggests that CRC has a unique VOC profile or signature that distinguishes it from other gastrointestinal pathologies. When this profile is combined with other (non-neoplastic) gastrointestinal disorders, the VOC signature is not sufficiently distinct to allow correct classification.

In the case of CRC vs different adenoma risk groups, the separation was again high. This implies that it is possible to separate malignant from pre-malignant disease based on urinary VOC signature patterns. When considering this as a basis for a screening test, the high specificity suggests the potential of using VOC-based analysis to reduce the number of unnecessary endoscopic procedures. This set of results warrant further exploration, to ascertain whether the separation seen is simply because the adenoma group represents another non-CRC group or whether there are changes specific to the VOC signature of colonic adenomas that make it more distinct from CRC than other groups.

To the best of our knowledge this is the first study to specifically examine adenoma detection by urinary VOCs. Applying FAIMS, the adenoma group showed poor separation from normal controls. Previous studies have reported on colonic adenoma detection by faecal [31] VOCs with low to modest test accuracy (sensitivity of 0.62). Advanced adenoma detection using breath VOCs [32, 33] demonstrated more encouraging results (sensitivity 1.0). These conflicting results suggest that there is more work needed to establish the mechanism of VOC signature changes in the presence of colonic adenomas and other gastrointestinal disorders. This is vital as adenomas represent a pre-malignant process with adenoma detection intrinsically linked to CRC mortality [34–36]. Adenoma detection is particularly pertinent to the BCSP population given the high adenoma incidence of approximately 50%, compared with around 15% in the average population [25, 37]. As faecal testing relies on the presence or absence of blood, it is poor at detecting adenomas, as most do not bleed [38]. If the patients with CRC and adenomas could be better identified using a urine test, this could revolutionise the screening process. Urine sampling has been demonstrated to be more acceptable to patients than faecal in the previous work by our group. In this study, recruitment was > 90%, far exceeding the FOBT uptake of approximately 60%.

The analytical technology associated with VOC detection is constantly being improved, with particular focus on refinement of data software and ion capture technique. The benefits of using FAIMS as the detection method of choice, have been described in the literature before [21], but the level of repeatability required for equipping the clinical setting with this technology has not yet been demonstrated.

This is the first reported study demonstrating the application of GC–IMS in CRC detection [39, 40]. GC–IMS have several advantages as a clinical tool. It is simple to use, meaning specialist training is not required. Also, it is portable, thus in theory, could be transported to the clinical area of need for instant analysis. Finally, due to the IMS component, it has the technology to isolate and identify the chemical compounds within the urine sample. This has the potential to broaden current understanding of CRC pathogenesis and also narrow the target window of chemicals that comprise the VOC profile in CRC. However, at this point the chemical National Institute of Standards and Technology (NIST) library required to perform this step is currently small.

It is important to highlight that the bowel screening patient group recruited for this study consisted of patients that had a positive FOBT. The authors recognise the importance of examining FOBT negative patients too when comparing a new screening tool with FOBT, but were constrained by the BCSP recommendations.

The current guaiac FOBT has poor selectivity for CRC, therefore, it would be interesting to repeat this experiment once the FIT is introduced for UK bowel screening. It has both superior sensitivity and specificity compared to the current FOBT. Combining VOC detection with FIT as a two-stage test in the asymptomatic population that forms the basis of a decision-making algorithm for further investigations, is an area of interest that may hold the most potential in the field of VOC diagnostics in CRC [41], rather than the use of VOC detection as a stand alone test. Further research could also test this algorithm (FIT plus urinary VOC profile) in the symptomatic population, where risk stratification is extremely difficult based on symptoms alone.

A limitation of this study was the small sample size for the CRC group, but this reflects the nature of the screening population with low CRC detection rates of around 8%. Machine learning algorithms that were used to analyse both sets of date always risk the possibility of overfitting of the data. This was minimised using a cross-validation technique and using two different technologies.

## Conclusions

Our results indicate detection of CRC and adenomas through urinary VOCs within a screening population is feasible. CRC can be correctly classified from control and adenomas using FAIMS and GC–IMS, but the classification of adenomas from control was poor. This approach to disease detection faces multiple challenges, that reflect the complexity of human disease and it is likely biomarker-led disease detection will require a panel of markers rather than focus on one specific marker in the screening population.

## Electronic supplementary material

Below is the link to the electronic supplementary material.

Supplementary material 1 (PDF 362 KB)

Supplementary material 2 (PDF 495 KB)
