# Supplementary material for: Colorectal cancer and adenoma screening using urinary volatile organic compound (VOC) detection: early results from a single-centre bowel screening population (UK BCSP)
Source: Tech Coloproctol. 2019 Apr 15;23(4):343–51. doi: 10.1007/s10151-019-01963-6 (PMC6536474; doi:10.1007/s10151-019-01963-6)

Journal: Techniques in coloproctology

Title:

Colorectal cancer and adenoma screening using urinary volatile organic compound (VOC) detection – Early results from a single centre bowel screening population (UK BCSP)

Authors:

Mozdiak E[1], Wicaksono AN[2], Covington JA[2], Arasaradnam RP[1]

Corresponding author:

Dr E Mozdiak, email: ella.mozdiak@nhs.net

***Schematic of the FAIMS analysis process** from sample introduction to ion count detection. (Schematic adapted from Owlstone, UK manual with permission)*

1. Vapour phase – the sample is introduced via a carrier gas to the ionisation region. Here components are ionised via a charge transfer process or by direct ionisation. Both positive and negative ions are formed.
2. The ion cloud enters the electrode channel, here a radiofrequency (RF) waveform is applied, creating a varying electric field under which the ions follow different trajectories depending on their mobility.
3. A direct current (DC) voltage, called a compensation voltage (CV) is swept across the electrode channel shifting the trajectories to the path of the detector allowing more ions to reach it. The detector then recognises both positive and negative ions.
4. The number of ions detected represents the concentration of chemical in the sample.

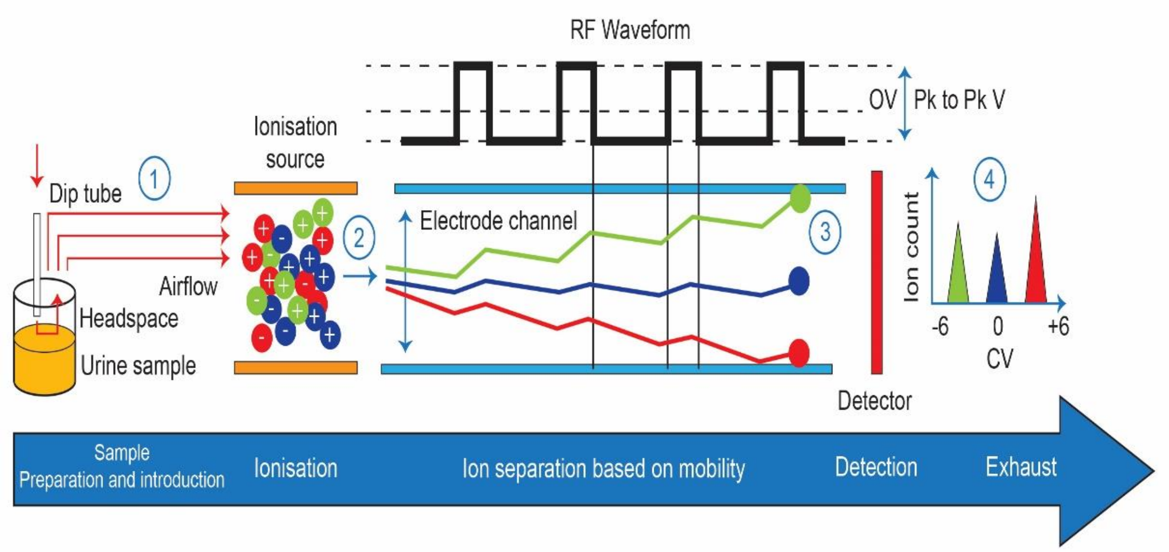

Supplement: Supplementary file 1 — Supplementary material 1 (PDF 362 KB) [file 10151_2019_1963_MOESM1_ESM.pdf]
