# Supplementary material for: Colorectal cancer and adenoma screening using urinary volatile organic compound (VOC) detection: early results from a single-centre bowel screening population (UK BCSP)
Source: Tech Coloproctol. 2019 Apr 15;23(4):343–51. doi: 10.1007/s10151-019-01963-6 (PMC6536474; doi:10.1007/s10151-019-01963-6)

Journal: Techniques in coloproctology

Title:
Colorectal cancer and adenoma screening using urinary volatile organic compound (VOC) detection – Early results from a single centre bowel screening population (UK BCSP)

Authors:
Mozdiak E[1], Wicaksono AN[2], Covington JA[2], Arasaradnam RP[1]
Corresponding author:
Dr E Mozdiak, email: ella.mozdiak@nhs.net

Raw VOC data analysis pipeline for FAIMS and GC-IMS.

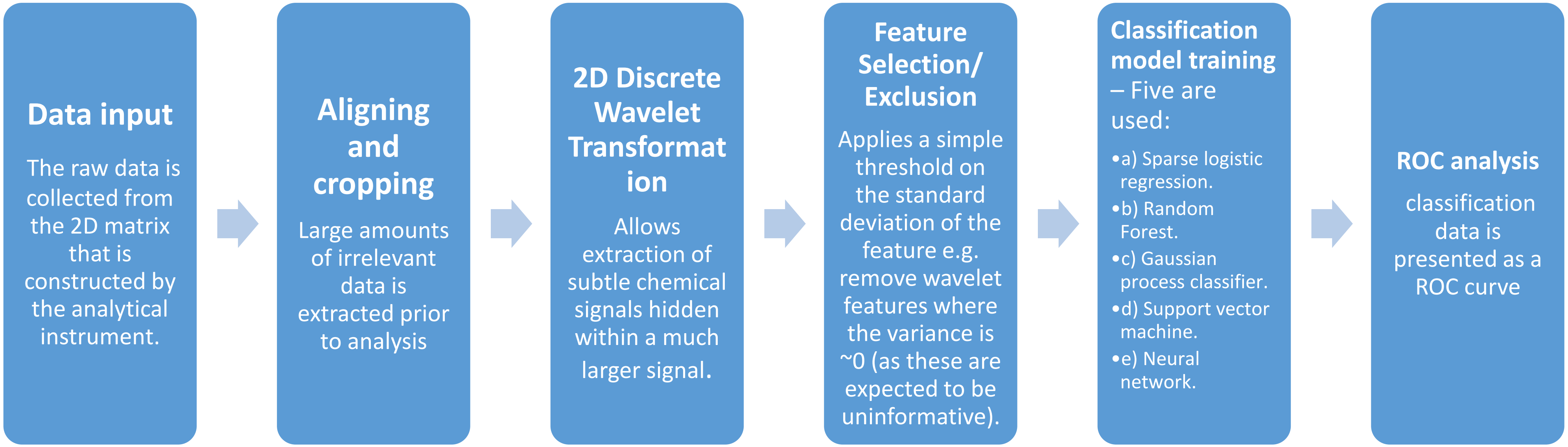

Supplement: Supplementary file 2 — Supplementary material 2 (PDF 495 KB) [file 10151_2019_1963_MOESM2_ESM.pdf]
